# UKCCCR Guidelines for the Use of Cell Lines in Cancer Research

**DOI:** 10.1054/bjoc.1999.1169

**Published:** 2000-04-03

**Authors:** UKCCCR

**Affiliations:** PO Box 123, Lincoln's Inn Fields, London, WC2A 3PX, UK


British Journal of Cancer (2000) **82**(9), 1495–1509
© 2000 Cancer Research Campaign
DOI: 10.1054/ bjoc.1999.1169, available online at http://www.idealibrary.com on IDEAL®

### UKCCCR Guidelines for the Use of Cell Lines in Cancer Research

UKCCCR, PO Box 123, Lincoln's Inn Fields, London WC2A 3PX, UK

Problems associated with cell culture are frequently ignored by the biomedical community, both in academic research and in the biotechnology and pharmaceutical industries. With depressing regularity, scientific data have to be retracted or modified because of cross-contamination between cell lines. Occult contamination with microorganisms (especially mycoplasma) and phenotypic drift due to serial transfer between laboratories are frequently encountered. Whatever the nature of the cell culture operation, large or small, academic or commercial, such problems can occur. The aim of these guidelines is to highlight these problems and provide recommendations as to how they may be identified, avoided or where possible eliminated.

The guidelines are meant to provide a series of pertinent and accessible reminders, which should be of benefit both to those for whom using cell lines is a new skill and those who may, despite years of experience, have allowed suboptimal procedures to become part of local practice. The guidelines are not meant to substitute for the many excellent textbooks which provide detailed information on many aspects of cell culture techniques and procedures.

Definitions of some terms frequently used in tissue culture are given in Appendix 1.

### SECTION 1: DERIVATION OF A NEW CELL LINE

### Ethical and legal issues

#### *Ethical approval for the use of human tissue*

These guidelines stress the responsibility of the researcher to the donor. Before any material can be collected from a hospital patient, ethical approval for the research must be obtained from the NHS Trust concerned. Therefore the Local Research Ethics Committee (LREC) should be consulted at an early stage. Grant-giving bodies usually will not fund projects involving patient material without the prior consent of the appropriate LREC.

The LREC form will usually request details of (a) the project (objectives, description, scale, duration), (b) those responsible for its supervision, sponsorship and the recruitment and selection of subjects, (c) the procedures and risks involved (both to the patients and those undertaking the research) and (d) the form of written consent to be obtained from the patient or relatives (see Appendix 2). The LREC must be informed of any involvement of a commercial company and the appropriate licensing of its products and equipment. LRECs have not usually been concerned with intellectual property rights but, with the increasing commercialization of research, this attitude may change. The NHS Trust will be interested in any commercial benefits arising from the research and usually will require that any additional costs incurred by the hospital as a result of the research are reimbursed.

Ethics committees are concerned that patient care and diagnostic needs are not compromised by the diversion of material for research purposes. It is recommended that clinicians are members of the team undertaking the study. Research may normally be conducted on tissue or specimens that are removed for the benefit of the patient and are surplus to diagnostic requirements. The removal of body fluids which are accessible with minimal invasion (e.g. blood, semen, urine, sweat, saliva) usually can be justified. However, if additional tissue is required, there must be a strong justification and the patient must be counselled accordingly.

#### *Informed consent*

The study of cells or DNA from patients has often, in the past, been regarded as an extension of the diagnostic process into research. It used to be accepted that the patient did not need to be consulted about the use of tissue surplus to clinical requirements. Attitudes have changed, and LRECs will generally require that the patient is asked for consent for tissue to be used for research purposes (see Appendix 2). In some circumstances (e.g. when additional tissue is required or a commercial waiver is requested), the LREC may require that the patient is informed in greater detail about the nature and purposes of the research.

Attitudes to the use of tissue from patients for research have also changed for another reason. Tissue derived from a patient may give rise, directly or indirectly, to a cell line or other product of commercial value. If the researcher wishes to control such financial benefit, then it will also be necessary to ask the patient to waive any right to the tissue or its exploitation.

Ethics Committees will need evidence that patient confidentiality will be maintained, including any personal data derived from the research, by coding the sample such that only clinical staff are aware of the patient's identity. If this confidentiality is to be broken, e.g. for familial studies, additional authority will be required.

*Correspondence to:* The Secretariat, UKCCCR, PO Box 123, Lincoln's Inn Fields, London WC2A 3PX, UK

These Guidelines were prepared for the UKCCCR during 1998 by an *ad hoc* committee comprising:
Dr J R W Masters (University College London, Chairman), Dr P Twentyman (UKCCCR Secretariat, London, Secretary), Dr C Arlett (MRC Cell Mutation Unit, Sussex), Mr R Daley (Sigma-Aldrich Company Ltd, Poole), Dr J Davis (Bio-Products Laboratory, Elstree), Dr A Doyle (Centre for Applied Microbiology and Research, Salisbury). Dr S Dyer (Medical Research Council, London), Dr I Freshney (University of Glasgow), Dr A Galpine (Cancer Research Campaign, London), Ms M Harrison (Imperial Cancer Research Fund, London), Dr H Hurst (ICRF Molecular Oncology Unit, Hammersmith Hospital), Dr L Kelland (Institute of Cancer Research, Sutton), Dr G Stacey (National Institute for Biological Standards and Control, South Mimms), Professor I Stratford (University of Manchester), Dr T H Ward (Paterson Institute for Cancer Research, Manchester)

### Ownership and patent rights

There is a long list of people and organizations who might lay claim to the ownership of specimens and their derivatives, including the donor and relatives, the surgeon and pathologist, the hospital authority where the sample was taken, the scientists engaged in the research, the institution where the research work was carried out, the funding organization supporting the research and any collaborating commercial company.

The ultimate control of subsequent ownership and patent rights will need to be negotiated between the research coordinator, the host institution, the funding organization and the commercial company. Most universities and research institutes have an office which deals with such negotiation, as do most of the larger funding agencies.

### Ethical approval for the use of animal tissue

It is essential that all legislation relating to the use of laboratory animals in scientific procedures is considered and that any necessary documentation is available, as described in the UKCCCR Guidelines for the Welfare of Animals in Experimental Neoplasia (1998). Some institutions have Animal Welfare Committees that act as Ethics Committees for research involving animals and specific approval from such a committee may be necessary.

### Material transfer agreements

Distribution of cell lines should be regulated by the originating laboratory or by a cell bank authorized by the originator. This will minimize successive transfers between laboratories and the resultant risks of cross-contamination and phenotypic drift (see Section 2). Ownership rights and exploitation should be covered in a Materials Transfer Agreement, signed by the host institution and the recipient before the cell line is transferred. The recipient should receive a signed statement indicating that ethical approval and informed consent has been received in respect of those cells. The donating institution may wish to state that no liability can be accepted for any problem arising from the use of the cells and that no guarantee of freedom from infective agents can be given. If a recipient of a cell line derives a subline by cloning and/or genetic manipulation, a new agreement of ownership will need to be established and this proviso should be contained in the Materials Transfer Agreement.

Restrictions on the use of cell lines should be minimal, but it is reasonable to insist on acknowledgement, and even co-authorship where the originating laboratory makes a substantial contribution to the subsequent work. However, merely supplying a cell line would not in itself normally warrant co-authorship of a paper describing work carried out using the line. The agreement should indicate that the cells must not be passed on to another laboratory or used for commercial exploitation.

### Authentication and characterization

Deriving a new human cell line is an expensive and time-consuming exercise. The subsequent value of the new cell line will depend on the ability to authenticate its origin and on the associated information that is available.

### Tissue

In addition to the tissue used to derive the cell line, it is also recommended for authentication that additional material is stored for:

a. CONFIRMATION OF ORIGIN. A small portion of the sample being used to originate the culture (or derived DNA or a blood sample) should be frozen or processed immediately for subsequent confirmation that any eventual cell line is derived from that patient (DNA fingerprinting or profiling is recommended for confirmation of origin)
b. CONFIRMATION OF DIAGNOSIS. A small portion of the sample being used to originate the culture should be removed and sent for histopathological confirmation. This is particularly important if the sample is supplied directly by the clinician, as it may not be representative of the tissue sent to the histopathologist for routine reporting – for instance it may be at some distance from a tumour and lack cancer cells
c. NORMAL TISSUE FOR COMPARISON. A small quantity of blood (e.g. 10 ml) or normal tissue should be frozen for comparative purposes (e.g. for analysis of loss of heterozygosity). This can also be used for authentication of the origin from that patient if necessary.

### Clinical information

As much of the following information as possible should be recorded, the first 4 items being of particular importance:

a. Age and gender of patient
b. Hospital and pathology numbers
c. Site of origin and nature of tissue specimen
d. Evidence of informed consent and waiver of commercial rights by donor
e. Histopathology report
f. Clinical history, treatment and subsequent course of disease
g. Additional information concerning stage of disease, tumour marker status and molecular genetics, etc.

### Accessory information

It is recommended that a complete record of the details concerning cell culture are kept, at least up to the point when the cell stocks are banked, including the type, sources and batch numbers of all media and additives and the methods by which the cell line was established. It is helpful to record the split ratios and the passage number.

A description of the cell type is helpful, e.g. epithelial-or fibroblast-like. When definitive characterization has been performed, this becomes epithelial or fibroblastic and the tissue type may be defined where specific markers are available. The transformation status of the cell line should be recorded as finite or continuous, normal or transformed and any special properties described such as growth capacity as a xenograft.

If a cell line has been genetically modified, it is necessary to describe the process used, including details of sequences and mode of insertion. For recombinant cell lines, additional information is needed, and additional tests are necessary to demonstrate lack of infectivity (e.g. following the use of retrovirus). For hybridomas, details of the sources of both sets of cells are needed. Where animal tissue is used to originate a culture, it is important to record the species and strain, age, gender and genetic status.

Although it may be necessary to use antibiotics in the primary culture, they should be removed as soon as possible and the cells tested for mycoplasma after a period of at least 2 weeks in antibiotic-free medium. The type of assay used for mycoplasma detection should be stated (see Section 4, Mycoplasma Contamination), as should the frequency and date of the last test.

© 2000 Cancer Research Campaign

*Cell line designation*

It is essential that the designation of the cell line is unambiguous. It should be unique and should maintain donor anonymity. The format could be as follows: Institution – Source or series – code or log number – clone number; e.g. MOG-G123-D4 (Medical Oncology Glasgow – Glioma Cell line 123 – clone D4). The full designation should be used in publications.

If the cell line is obtained from another source, its original designation must be retained. If obtained from a cell bank, its accession number should be quoted in publications. Genetic modifications, sublines and clones should be indicated by a suffix, following the original designation. It is important that the designation is unique so that there is no ambiguity with other cell lines or biological resources during literature searches.

*Publication*

All first publications should include all the information described in the previous sections and subsequent publications should cite the first publication. Every publication should confirm that the cultures have been tested for mycoplasma using a sensitive method, and confirming that the test is negative. The first publication should also provide evidence that the cells have been derived from the individual claimed to be the source. Some journals insist on cell lines being made available as a condition of publication, so that other laboratories can repeat the work. Information on deposits in cell banks or whom to contact to obtain cells is helpful in this regard. Publication of work with the cell line implies its entry into the public domain and the right of others to acquire the cell line from the originator or the nominated cell bank.

### SECTION 2: CELL LINE ACQUISITION

Whilst the problems of cross-contamination, microbial contamination and phenotypic drift can occur in the laboratory of origin, these are more likely to arise during the serial transfer of cell lines among other laboratories. It is therefore necessary to make appropriate checks whenever a new cell line is acquired.

### Checking a cell line new to the laboratory

Characterization is essential not only when deriving new lines, but also when a cell line is obtained from a cell bank or other laboratory. A published description of a cell line with a certain property is no guarantee that it is still the same line or has the same properties. Enormous amounts of time and effort have been wasted by scientists using cross-contaminated cell lines which are either of a different species or cell of origin to that claimed, or cell lines which are contaminated with mycoplasma or some other microorganism.

*Quarantine*

Cell lines new to a laboratory should be quarantined; i.e. kept entirely separate from existing cell line stocks. Ideally, a separate quarantine laboratory should be available for this purpose. The next best approach is to have a Class II microbiological safety cabinet (MSC) and an incubator dedicated for quarantine. If this is not possible, other steps should be taken to minimize the risk of contamination, including (a) cells in quarantine should be handled only after all the other cell culture has been completed that day, (b) the new cultures should be placed in a dedicated incubator or a sealed container before going into a general incubator, (c) the MSC should be cleaned after use with a suitable disinfecting agent (such as 5% Dettol in 70% ethanol) and run for at least another 5 min prior to shutdown.

Cells should be quarantined until the origin of the cells has been authenticated or a DNA fingerprint or profile defined and it is confirmed that they are negative for microorganisms.

*Characterization*

DNA fingerprinting or profiling are the recommended methods for confirming the origin of a cell line and to check for cross-contamination between cells. Ideally it will be possible to compare the DNA with that of the tissue of origin. Unfortunately this is only possible in a minority of the cell lines already available. Nevertheless, it is desirable that a DNA fingerprint or profile is defined before the cell line is used, so that at least it can be distinguished from other cell lines in the same laboratory and other common cross-contaminants and can, therefore, be tracked through subsequent transfers.

As a minimum safeguard against microbial contamination, screening for mycoplasma is essential before the cell line leaves quarantine.

Where possible, new cell stocks should be characterized for features which will enable monitoring of genotype and phenotype variability. These include the karyotype (by G-banding or fluorescence in situ hybridization) and production of cell or tissue specific markers (e.g. cell surface markers and intermediate filament proteins such as cytokeratins for epithelial cells, glial fibrillary acidic protein for astroctyes, prostate-specific antigen, etc.).

It is helpful to measure under defined conditions features such as population doubling time, colony forming efficiency, morphology under phase contrast during both exponential and plateau-phase growth (with photographs) and the histopathology of xenografts in immune-deficient mice.

### Sources of cell lines

Acquisition of cell lines presents a number of potential hazards. Cell lines may simply not be what they are claimed to be. In the past, many cell lines have been passed from laboratory to laboratory with a range of labels and turned out to be something completely different. This is still happening with unnecessary frequency. Human cell lines may carry viral contamination such as hepatitis or HIV, representing a health hazard to laboratory workers (see Section 3, Safety). They may be contaminated with bacteria, fungi, mycoplasma, or viruses, which may spread to other cell lines.

The more laboratories that a cell line has passed through since its origin, characterization and contamination testing, the less reliance should be placed on its documented properties. However, even the originator as source is not a guarantee of authenticity. If the receiving laboratory wishes to place any reliance on historic data obtained with a cell line, it should always carry out its own testing procedures (as described in Section 2, Characterization) before accepting an incoming cell line into general use.

*Cell culture banks*

A number of ‘culture collections’ or ‘cell banks’ have been established by either academic or commercial bodies (see Appendix 3 for web sites). The prototype was the American Type Culture Collection (ATCC) currently holding over 14 000 cell lines. Other major collections are the European Collection of Animal Cell

© 2000 Cancer Research Campaign

Cultures (ECACC) with over 1500 cell lines, the German Culture Collection (DSMZ), Coriell Repositories, Camden, NJ and the Japanese Culture Collections (RIKEN and JCRB). Cell lines from these sources are unlikely to be contaminated with microorganisms, unless so stated in the accompanying literature. However, some of these cell lines have been acquired following multiple transfer between laboratories, so authenticity is not guaranteed unless specifically stated.

### SECTION 3: CELL LINE PRACTICE

Detailed information on methodology can be found in a number of text books, including *Basic Cell Culture* by John Davis, IRL Press, 1994 and *Culture of Animal Cells* by Ian Freshney, 3rd edition, Wiley-Liss, 1994.

### Safety

Cell culture in the commercial sector is subject to regulation. For example, where cell culture products are to be used by the pharmaceutical industry, Good Manufacturing Practice (GMP) (HMSO, 1997) must be complied with, along with the more specific guidance contained in a number of other documents issued by the European Union, the US Food and Drug Administration and the International Conference on Harmonisation (ICH). These guidelines on cell culture are in addition to local and national safety regulations, and do not replace rules of safety within individual laboratories, as these vary according to local circumstances. The advice of the local Biological Safety Officer should be sought where there is any doubt about the introduction of new materials or procedures.

Employers are responsible for employee safety under the Health and Safety at Work Regulations by providing information, instruction and training and effective protection against hazard in the workplace. The most relevant component is the COSHH (Control of Substances Hazardous to Health) regulations (Health and Safety Commission, 1994). These regulations foster safe working practices by establishing that any proposed procedure is both justifiable and safe by requiring that a risk assessment is made before work is started. The COSHH regulations also set out a duty for employees to collaborate fully so that employers can meet the legal obligations. The risk assessment must be approved by the local authorized Biological Safety Officer. It should deal with the entire process and not just individual hazardous chemicals and biological agents. Risk assessments should not be copied from one laboratory to another since the same hazards represent different risks according to local conditions and the scale of the operation.

The main safety hazard arising from cell culture is from agents carried either by the cells or from the components of the culture medium. Cells can carry viruses and at least one fatality due to a viral infection acquired from cells has been reported (Hummeler et al, 1959). Serum could also contain a variety of microorganisms, including viruses and mycoplasma.

Using body fluids or cells derived from laboratory staff for research purposes is not recommended. The use of blood or tissue from laboratory staff for the development of transformed cell lines is prohibited, as the person concerned would have no immunity to the transformed cells.

#### *Clinical specimens*

Advice on dealing with blood and HIV-infected material is contained in the guidelines from the Health and Safety Executive (HSE) (HMSO, 1996). Material with a high potential risk of infection should be excluded or handled appropriately. All samples of blood, body fluids, secretions, tissues and cells are potentially infectious. Risk of exposure to infection can be minimized by avoiding the use of 'sharps' (such as needles and blades) and any items or processes likely to create aerosols. When taking blood, the needle should be removed from the syringe and discarded safely into a 'sharps' container, before the specimen is transferred.

#### *Primary cultures*

There are documented cases of serious laboratory-acquired infections (e.g. hantavirus, lymphocytic choriomeningitis virus) from tissue, primary cell cultures and tumour cells taken from, or transplanted into, rodents (Lloyd and Jones, 1984). When obtaining primary tissue from laboratory animals it is important to ensure that the animals used are free of specific pathogens and suppliers should provide evidence of testing. This information should be used in risk assessments and cross-referenced in laboratory record books where the respective primary cells are used.

#### *Continuous cell lines*

The extensive use of continuous cell lines indicates that there is little risk from routine cell culture. However, since most cell lines are not fully characterized it is wise to regard all such material as potentially infectious. A tumour grew in a laboratory worker accidentally inoculated with cells of a human tumour cell line through a needle (Gugel and Sanders, 1986) and cancers have been transferred between people during transplantation (Southam, 1958). Although the growth of tumour cells from a different person is unlikely in healthy individuals, anyone with a compromised immune system is at greater risk.

#### *Genetically modified cells*

The introduction of genes can reactivate dormant infectious agents in the host cell or create new agents by recombination. Viral vectors that can infect human cells (e.g. amphotropic retroviruses) are particularly dangerous. Recommended procedures for creation, use, storage, transportation and disposal of genetically modified organisms, including modified cell lines, are given in the Genetically Modified Organisms (Contained Use) Regulations (Health and Safety Commission, 1992) (NB: These do not apply to construction of somatic cell hybrids). These regulations describe how to make a full risk assessment which must receive approval from the Local Genetic Modification Safety Committee, and in certain cases specific approval from the HSE may be required.

Genetically modified cells may require special conditions. For example, selective pressure may need to be maintained on transfectants to retain the genetic modification, and the pressure may need to be maintained during storage. Distribution of genetically modified cells may be subject to regulation, depending on the modification.

#### *Containment*

Containment level 2 is the minimum requirement for manipulating human cancer cell lines and is described in the Advisory Committee on Dangerous Pathogens (ACDP) guidelines (HMSO, 1995). This level of containment is also applicable to untested cell products, such as monoclonal antibody-containing supernatants or

 © 2000 Cancer Research Campaign ascites and cell pastes. These ACDP guidelines also recommend that all subculture, or other procedures involving the manipulation of bulk cells, should be performed in a Class II Microbiological Safety Cabinet (MSC). Laminar flow devices other than MSC should not be used for cell culture. Horizontal flow cabinets, where the airflow is directed at the operator, are particularly hazardous and must never be used when working with cells or potentially infectious cell derivatives.

The spread of infection often occurs via contaminated aerosols and any process which produces aerosols from crude cell culture preparations is a potential source of infection. Such processes (e.g. centrifugation, tissue disaggregation, vortex mixing) should be contained or the material rendered harmless before it is processed. Special guidelines for the safe use of flow cytometers with unfixed cells have been published by the International Society for Analytical Cytology (Schmid et al, 1997).

### *Disposal*

Control of the disposal of laboratory waste should prevent exposure of staff and environment to infectious hazards and prevent contamination. The Environmental Protection Act 1990 and the Environmental Protection Act Part II, The Special Waste Regulations 1996 (copies available from The Stationery Office, PO Box 276, London SW8 5DT) define clinical waste and appropriate procedures for its collection and disposal. Those producing clinical waste (including drugs, pharmaceuticals, animal and human material and any items contaminated with these materials) have a duty in law to ensure its safe disposal. All infected waste arising from work in laboratories should be made safe to handle by appropriate means (e.g. autoclaving), before disposal by incineration. Details of recommended procedures are given in HSE guidelines (Health Services Advisory Committee, 1992).

### Training

Trainees need to learn the theory, dangers and safety measures of cell culture before starting in the laboratory. Practical training is best carried out on a one-to-one basis, with extensive reference made to any relevant Standard Operating Procedure (SOP). As compliance with any demanding technique tends to decrease with time and familiarity, performance should continue to be monitored.

Individuals experienced in cell culture starting in a new laboratory should read the protocols specific to the laboratory, such as safety, waste disposal, autoclaving, incubator use/sharing, labelling of cultures and medium storage.

### Storage and banking

The first step to ensuring a reliable and reproducible supply of cells is the establishment of a Master Cell Bank of 10–20 ampoules. One ampoule from the Master Cell Bank is used to generate a Working Cell Bank or Distribution Cell Bank (Stacey and Doyle, 1997). The Working Cell Bank contains sufficient ampoules to provide at least one ampoule for every 3 months of the proposed experimental period plus sufficient ampoules for contingencies and distribution. Incorrect or serial banking (as occurs for cultures passed from one laboratory to another in a chain) results in a progressive increase in the population doubling number and additional risk of contamination or loss of key characteristics.

### *Cryopreservation*

Automatic controlled-rate cooling apparatus provides the most reproducible cryopreservation. Commercial equipment designed to fit in the neck of a liquid nitrogen freezer and reduce the temperature by approximately 1°C a minute is usually effective, and homemade devices (e.g. expanded polystyrene boxes packed with paper towelling) placed in a –70°C freezer overnight can also be used successfully. Dimethyl sulphoxide (DMSO) at 5–10% v/v is the most common cryoprotectant used for mammalian cells. However, it can be toxic and may cause differentiation in some cultures (e.g. HL-60). Glycerol (10–15% v/v) may be a suitable alternative. Every time a batch of cells is frozen down, it is recommended that one vial is resuscitated immediately to check viability. Vials removed from the bank should be thawed rapidly (by immersion in a water bath at 37°C) and the cell suspension diluted in stages with prewarmed medium.

### *Storage*

Cell stocks should be kept below –130°C as viability may be progressively lost within a few months at –80°C. For liquid nitrogen storage it is a legal requirement in the UK to store potentially infectious material in the vapour phase. This reduces the risk of transfer of contaminating organisms (Tedder et al, 1995) and eliminates the hazard of liquid nitrogen penetrating ampoules which may then explode on warming. For security, important material (e.g. Master Cell Banks) should be divided into more than one storage vessel, preferably on different sites.

Hazards associated with the use of liquid nitrogen include frost bite, asphyxiation (i.e. oxygen depletion) and risk of infection and injury due to explosion of ampoules. Plastic ampoules are preferred as glass ampoules are more likely to explode. The storage area should be well ventilated and where large numbers of liquid nitrogen vessels are involved there should be an oxygen deficiency alarm and mechanical ventilation (preferably activated through the oxygen monitor). Appropriate personal protective garments (e.g. insulated gloves and face masks) and equipment for safe manual handling of nitrogen vessels should be available. Staff should also receive training in safe working practices for the nitrogen storage facility. Access to storage vessels should be strictly controlled.

Freezers should be fitted with alarms and storage temperatures regularly checked. It is recommended that levels of liquid nitrogen in the storage vessels are recorded at least once a week. Periodic audits for evidence of regular maintenance, monitoring and stock control will also help ensure safety and security of storage facilities.

### Culture reagents

It is recommended that reagents and sera are purchased from suppliers who issue certificates of analysis or results of quality control (QC) testing with each batch of products. Reagents may be purchased in bulk to avoid variation between batches, depending on shelf life. Serum should be stored at –20°C, but not in frost-free freezers as temperature cycling may crack the bottles. The shelf life of serum is 12–18 months and longer term storage is not recommended as any advantages gained by a single batch may be offset by deterioration. The shelf life of single strength medium is approximately 9–12 months, concentrated medium (10×) approximately 12–24 months and powdered medium approximately 24–36 months.

© 2000 Cancer Research Campaign

### *Media production*

The production of media from individual ingredients is complex and time-consuming. Most commercial suppliers offer a custom media service for specialized formulations. Most basic media formulations are offered both as single strength and as 10× concentrated liquids by suppliers. Considerable cost savings can be achieved by using the 10× concentrates. If concentrate is used, this is diluted into bottles containing sterile high quality water. Sterile L-glutamine and sodium bicarbonate are then added and finally the pH is adjusted. The advantage of this system is that it is quick and technically undemanding. However, several points should be borne in mind.

a. Media concentrates have changes made to their basic formulations, mainly to overcome problems of solubility
b. Precipitate is often seen on storage. If the concentrate is aliquotted the precipitate can cause variation between bottles
c. Suppliers acidify the medium to improve solubility. This in turn requires significant amounts of base to neutralize the medium.

### *Powdered media*

Powdered media produce more stable uniform products with longer shelf lives than concentrates. However, the process does require specialized equipment for filtration and bottling. The following general points should be noted:

a. The powder should be free flowing and white to off-white in colour, with no sign of dampness
b. The medium should be stirred until all the powder is dissolved. The presence of fine particulate matter may require pre-filtration or a change of supplier
c. Medium should always be prepared and filtered on the same day.

Sterilization requires filtration to a pore size of 0.22 μm. Cellulose filters are most common but polyvinylidene fluoride (PVDF) filters should be used when protein is present in the medium. Although a 0.22 μm filter will prevent the passage of bacteria and fungi, mycoplasma can pass through pores of greater than 0.1 μm in diameter. It is probably easier to screen cultures regularly for mycoplasma contamination than to try to exclude it from a source such as media. It may be possible to exclude viruses by ultracentrifugation, but this is impractical in most laboratories. Single-use disposable cartridges are recommended as the most convenient option for media filtration. The following basic points should be noted:

a. The equipment should be dedicated for media production only
b. A Class II MSC should be dedicated to media and supplement production. If this is not possible then the cabinet should not have been used for cell culture for at least 1 h. It must also be cleared of all equipment and thoroughly cleaned with 70% alcohol or non-corrosive disinfectant
c. All tubing should be clean and autoclaved before use and connections should be securely in place
d. Sterile bottles and caps should be stacked outside the cabinet and introduced one at a time to receive medium. Stacking bottles within the flow cabinet will seriously compromise the airflow, and consequently sterility
e. During bottling, representative samples should be drawn off at regular intervals. These samples should then be incubated at 37°C for at least 10 days to check for contamination
f. Most bottled media should be stored at 4°C in the dark
g. If any sample shows contamination, the whole batch of medium should be discarded.

### *Serum batch testing*

Simple preliminary tests can help avoid the disastrous consequences of using media, sera or supplements which do not adequately support cell growth. Batch testing of serum should use a range of cell lines and may include criteria for (a) cell attachment and spreading, (b) cloning efficiency, (c) growth rates and where appropriate (d) a functional assay. Low serum concentrations (e.g. 1%) can help highlight differences between sera. It is important to limit carry-over of the old serum during testing, as this could mask differences between the old and new batches. Large batches of serum should be purchased when possible and retested before use.

### Record keeping

Details of all routine and experimental procedures should be recorded as they are generated. Good practice requires that records must be dated, legible, clear in content and made in ink directly into a bound laboratory notebook or onto a standard form. Enough detail must be recorded to enable the work to be reproduced exactly. Standard Operating Procedures (see below) should be referenced wherever possible. Graphs, figures and photographs should be attached to the notebook and be signed, dated and identified in such a way that, should they become detached, they could be re-assigned to their correct place. If large numbers of print-outs or other documents are generated, these should be annotated and stored in a dedicated file.

Records of routine procedures carried out, such as cell counts, cell line passaging and medium preparation can be kept on standard forms designed for the purpose. These should be stored in a dedicated file, and cross-referenced in experimental notebooks as required.

A certificate of analysis should be requested from the supplier for each batch of material, and this should be stored securely for future reference with the date received.

The originals of all experimental records remain the property of the funding agency or laboratory, and must be lodged with them when an experimenter leaves that laboratory or changes funding agencies. Such records should be securely archived, with systems in place to permit easy retrieval along with protection against tampering.

### *Standard operating procedures*

Procedures that are regularly carried out in a standard manner are best documented in the form of Standard Operating Procedures (SOP). This is a clear and detailed list of instructions, written such that suitably trained individuals can understand and perform the task in the intended manner. It should include details of the equipment, reagents, and techniques to be used, as well as methods for calculating and interpreting the results. Ideally, each laboratory or organization should have its own system for the issue and tracking of SOP. This should ensure that all copies can be tracked so that all scientists have the most recent versions, and that they are reviewed on a regular basis (at least once every 2 years is suggested). A

© 2000 Cancer Research Campaign nominated scientist should be responsible for the issue of all SOP, such that only one approved version is current, reflecting best practice. Ideally, the system should include provision for permanent archiving of all versions and revisions of all SOP.

### Equipment

### *Microbiological safety cabinets*

Most cell culture is undertaken in a Class II MSC. These provide protection to the operator and environment whilst maintaining a clean working environment, but give no protection against toxic, radioactive or corrosive materials. The effectiveness of a MSC is dependent on its position, correct use and regular testing.

Cabinets should be sited away from doors and through traffic. Movement in the area of the MSC will disturb the airflow and therefore access to the area should be restricted to essential personnel. Recommendations for siting MSC are given in BS5726 Part 2 (HMSO, 1992).

Class II cabinets which are used for genetically modified organisms or primary human material should be tested every 6 months for airflow and filter integrity and annually for operator protection. Cabinets used for general cell culture should be tested annually. Testing and servicing should be carried out by trained competent personnel. Before servicing and testing is carried out, adequate fumigation is required. This is usually performed using formaldehyde gas. Training is essential before this procedure is carried out. An equipment safety certificate is normally required by servicing engineers before testing can begin.

When performing cell culture work within a MSC it is important to minimize the potential for contamination of the working environment and cross-contamination between cultures. This can be greatly assisted by the following:

a. Do not make rapid movements within the cabinet, since this may disrupt the airflow
b. Manipulate fluids slowly and gently with the assistance of a pipetting aid to avoid the creation of aerosols
c. Never have more than one cell line at a time in the cabinet
d. Do not overcrowd the cabinet and never obstruct the front opening and grille
e. Organize the work area such that sterile reagents and cultures do not come in contact with each other
f. Use caution when homogenizing tissues or cells in a MSC. If high energy processes such as sonication are used the particles cannot always be assumed to be contained by the cabinet airflow
g. Clean and decontaminate the cabinet inner surfaces after each work session and periodically decontaminate the tray under the MSC working surface
h. When working in a MSC, a Bunsen or similar burner must not be used (unless absolutely required for a specialized procedure) as they disrupt the airflow pattern, thus reducing the cabinet's effectiveness, and they pose a fire risk.

### *Incubators*

Most modern incubators are humidified and used with an atmosphere typically containing 5% carbon dioxide. The following points should be considered:

a. Humidifying water should contain an antibacterial/antifungal agent as per manufacturer's recommendations.
b. Incubators should be calibrated for temperature and gas composition
c. Carbon dioxide levels should be checked monthly using a Fyrite apparatus (marked deviations will be evident as a change in pH of the medium)
d. Every 6–8 weeks the incubator should be emptied, dried and cleaned with 70% alcohol or equivalent non-corrosive disinfectant. All shelves should be similarly removed and cleaned
e. Individual trays on which culture flasks can be easily moved in and out of the incubator should be used. These may be cleaned more frequently and help reduce contamination from spillages
f. Incubator temperatures and contents should be inspected daily
g. Spillages must be dealt with immediately
h. All infected plates, dishes or flasks must be removed immediately and disposed of appropriately
i. Incubators must only be used for cell culture and not for incubating microorganisms or biochemical samples
j. Gassed incubators should be attached to a suitable cylinder change-over unit
k. Cylinders used to supply gas should be securely anchored
l. Cylinders should be clearly labelled and have the correct regulating valves attached
m. Cylinders should be changed by trained personnel wearing suitable high impact eye protection.

### *Autoclaves and sterilizing ovens*

Autoclaves are used for sterilizing equipment and consumables. Correct function and safe operation are described in the HSE guidelines (Health and Safety Executives, 1990). Autoclaves must be covered by insurance, which will necessitate an annual inspection. It is essential that proper protective clothing (including a face visor and heat-proof gloves) are used, and the autoclave not opened until the temperature has fallen below 50°C. Autoclaving of liquids in glass containers can present particular hazards.

It is essential that regular checks are made to ensure that the autoclave is operating at the required temperature and pressure. Qualitative indicators (e.g. autoclave tape) are useful to distinguish items that have been autoclaved from those which have not. They do not, however, provide any guarantee that a full autoclave cycle has occurred and hence that the item is satisfactorily sterilized.

### *Water purifying apparatus*

The use of high purity water is essential for successful cell culture. Reverse osmosis followed by passage through mixed bed ion exchange resins and carbon and microspore filtration provides pyrogen-free water of tissue culture grade. Water should be measured for pH and conductance and can be tested commercially. Serum can protect cells from toxins and consequently the use of high purity water is critical in low protein or serum-free conditions. The purity of water is only maintained if it is placed in suitably clean bottles dedicated to storage of water or media.

### SECTION 4: CELL LINE PROBLEMS: IDENTIFICATION AND ELIMINATION

Cultures should always be examined under an inverted phase microscope before any manipulations are performed, and frequent assessments should be made of the viability of the cell population.

© 2000 Cancer Research Campaign

### Cell line cross-contamination

Whenever a rapidly-growing, continuous cell line is maintained in a laboratory there is a risk that it may cross-contaminate other, more slowly-growing lines. There is a long history of this problem, highlighted in the 1960s and 1970s (Gartler, 1967; Lavappa, 1978), but now often ignored. Few authors using cell lines such as KB, Chang liver, or Hep-2 acknowledge that they are cross-contaminated with HeLa cells, and probably carry none of the original cell lineages. Similarly, some human breast and bladder cancer cell lines with a variety of names are in fact MCF-7 and T24 respectively. This may have limited importance if the property under examination is cell line-specific and intrinsically important, but if comparisons are to be made between cell lines or if extrapolations are made to the tissue of origin or a particular class of cell lines, then interpretation of the data can only be made if the target cell lines are correctly identified.

### *Identification of cell line cross-contamination*

The advantages of the methods available for determining the origin and uniqueness of a cell line are listed in Appendix 4. It is recommended that a reproducible DNA fingerprinting or profiling method is routinely adopted for all cell lines within the laboratory.

### *Prevention of cell line cross-contamination*

A problem arises when a small number of cells from a rapidly growing cell line are inadvertently transferred into a culture of more slowly growing cells. Such transfer can occur by a variety of routes such as the accidental touching of a pipette on the neck of a bottle of medium or some other common reagent, or by the presence of an aerosol in the MSC at a time when flasks are uncapped.

In a theoretical example, a single cell from a line with a population doubling time of 12 h is transferred into a culture of $10^5$ cells of a cell line with a population doubling time of 48 h. By the time the slow-growing cell line has expanded from $10^5$ to $3.2 \times 10^6$ cells (i.e. 5 doublings or 10 days), the rapidly growing cells will have doubled 20 times (i.e. from a single cell to $10^6$ cells), and hence comprise nearly 25% of the total cell population. After one further passage, the rapidly growing cells will predominate.

Because of the problem of cross-contamination, derivation of new cell lines should allow for future authentication by storing samples of tissue or DNA from the source individual for subsequent DNA fingerprinting. Cell lines new to a laboratory should be developed into frozen stocks and typed. Change in cell behaviour or morphology may indicate a cross-contamination and constant vigilance and attention to good tissue culture practice are essential.

Simple precautions must be taken to minimize the possibility of cross-contamination, including:

a. Only one cell line should be used in a MSC at any one time. After removal of the cells from the cabinet, it should be swabbed down with a suitable liquid disinfectant and the cabinet run for 5 min before the introduction of another cell line
b. Bottles or aliquots of medium should be dedicated for use with only one cell line
c. The formation of aerosols must be kept to a minimum
d. Regularly return to frozen stocks (except where essential, never grow a cell line for more than 3 months or ten passages, whichever is the shorter period)
e. All culture vessels must be carefully and correctly labelled (including full name of cell line, passage number and date of transfer), as must storage containers.

### Mycoplasma contamination

Mycoplasmas are small, self-replicating prokaryotes (0.3–0.8 μm diameter), lack a cell wall and have the ability to cytoadsorb onto host cells. Mycoplasma is one of the most serious forms of cryptic contamination and its presence is not detected unless appropriate tests are made or until some aspect of cell behaviour is noticed to have changed. The consequences of mycoplasma contamination have been documented (McGarrity et al, 1984), influencing almost every aspect of cell biology. Between 15 and 50% of cell lines submitted to cell banks are contaminated with mycoplasma. Laboratories which do not test for mycoplasma probably harbour contaminated cell lines and may even have their entire stocks contaminated, as mycoplasma spreads readily among cell lines via reagents and media, the operator and the work surface. The presence of mycoplasma may invalidate the results obtained with that culture. The presence of mycoplasma-infected cultures can result in the shut-down of the entire laboratory until the infection can be eliminated, whereupon complete restocking is required.

### *Identification of mycoplasma contamination*

The origin of contamination is usually traced to mycoplasma present in animal (bovine) serum or to human oral mycoplasma transferred by droplet infection during cell culture. Methods for detecting mycoplasma species are summarized in Appendix 5.

The simplest test for the detection of mycoplasma in cultures is the use of a fluorescent dye which binds directly to DNA causing fluorescence (e.g. Hoechst 33258) which can be seen by fluorescence microscopy. Mycoplasma-positive cells will show intense fluorescent spots on the plasma membranes or show filaments which may be absorbed onto the cells. Uncontaminated cells show only brightly fluorescent cell nuclei. The technique is rapid (less than 30 min), but requires heavy contamination ($10^6$ mycoplasma $ml^{-1}$) to produce a clear positive result. If however, the suspect cells are co-incubated for 2–4 days with an ‘indicator’ cell line (such as 3T3) which is particularly suitable for demonstration of positive staining, then sensitivity can be substantially increased.

Microbiological culture techniques are available that operate at a greater sensitivity, but it can take up to 21 days to obtain a result, a positive control is needed, and the result may require expert interpretation. A variety of polymerase chain reaction (PCR) based methods are available, some of which have been utilized as commercially available detection kits. It is recommended to use a combination of DNA staining and a PCR-based method once every 3 months for all growing cultures in the laboratory and for every new cell line as it enters the laboratory. In addition, all Master and Working Cell Banks should be tested at the time of freezing.

### *Prevention of mycoplasma contamination*

Quality control and good working practice will reduce potential problems. It is important that frozen stocks are created immediately after testing and re-tested before distribution. If cells are cultured for more than 3 months after testing, they should be re-tested. Regulatory bodies now insist that cell cultures used for the production of reagents for diagnostic kits or therapeutic agents are free from mycoplasma infection. Also, some scientific journals

© 2000 Cancer Research Campaign have the policy of requiring statements from authors that the culture work reported in those journals is carried out with mycoplasma-free cells.

Normally, when contamination with mycoplasma is apparent, the recommendation would be to discard the cultures and start again. If necessary, and only if the contamination is not extensive, then it is often possible to rescue the cells by treatment with one of the commercially available antibiotics. This must only be considered for a remedial action, not as a routine supplement to growth media (and thereby a substitute for good cell culture practice).

### Contamination by other microorganisms

With correct working practice it is not necessary to use antibiotics when working with established cell lines, and the use of antibiotics should be discouraged. Microbial contamination may be overt, and hence act as a signal to discard the culture, but if antibiotics are used, contamination may be repressed but not eliminated. Such cryptic contamination may co-exist with the cell culture and only appear when the culture conditions change, or a truly resistant organism appears.

A cautionary note is that antibiotics and antifungal agents act by inhibiting biochemical functions of the organism, and consequently may alter the outcome of experiments. For example, amphotericin B is a membrane active agent and may therefore interfere with any mammalian cell experiments involving membrane trafficking or intercellular signalling.

### *Bacteria and fungi*

If cells are cultured in antibiotic-free media as recommended, contamination by bacteria, yeast or fungi can usually be detected by an increase in turbidity of the medium and/or a decrease in pH (yellow in media containing phenol red as a pH indicator). It is recommended that cells are inspected daily, and must always be examined under an inverted phase microscope before use in an experiment.

The two methods generally used for bacterial and fungal detection are microbiological culture in special media and direct observation using Grams stain. It is recommended that the help of a hospital microbiology laboratory is sought with identification and sensitivity testing.

If a cell culture is contaminated with bacteria or fungi, then the best method of elimination is to discard the culture and obtain fresh stock cultures or new supplies. In the case of irreplaceable stocks, it will be necessary to use antibiotics. The more antibiotics that are tested, the more chance there is of finding one that eliminates the infection. However, if the cells have been routinely grown in media supplemented with antibiotics (which is not recommended), it is almost certain that the contamination will be with organisms that are already resistant to this and some other antibiotics.

To eliminate infection, the cells should be cultured in the presence of the antibiotic for at least three passages. If the contamination appears to be eradicated, then the cells should be cultured in antibiotic-free medium for 1 month before re-testing.

### *Viruses*

As long as serum is used to supplement media and natural trypsin is used in subculture, there will always be a risk that endogenous infections in the source of the reagent will infect the culture. The source of viral contamination can be from the tissue from which the cells are derived (e.g. HIV from Kaposi's sarcoma cells, EBV from lymphoma cells). Alternatively, contamination can be derived from growth media from other infected cultures or, as a more remote possibility, from laboratory personnel. Another route of infection can be during passage of cells in experimental animals. This is important when considering the use of cell lines for implantation of xenograft tumours. Not only do the cells to be implanted need to be free from contamination by extraneous viruses, but also the animals into which the transplant is to be made should not harbour viruses that could affect the growth and response to therapy of the cells under study (UKCCCR, 1998).

As with mycoplasma, elimination is difficult. However, what is worse, there are no simple universal diagnostic tests to identify viral contamination. To identify viruses necessitates screening with a wide panel of immunological or molecular probes. As yet, such testing is restricted to human pathogens such as HIV and hepatitis B, and few laboratories screen for animal viruses on a routine basis.

### *Transmissible spongiform encephalopathy*

Transmissible spongiform encephalopathy (TSE) (including what is known as bovine spongiform encephalopathy, BSE or mad cow disease) is unlikely to be present in cancer cells or tissue culture products. However, a risk of exposure must be regarded as potentially present, particularly since all the routes of transmission have not been identified. Some regulatory authorities now demand that serum used in the production of pharmaceutical, veterinary, and sometimes diagnostic products can only be obtained from specified countries of origin where BSE has not been diagnosed. Some countries, including the USA, will only allow import of cells that have been cultured in media containing serum from BSE-free areas.

### Genetic instability and phenotypic drift

Two other major problems which can affect the utility of cell lines are genetic instability and phenotypic drift. Wherever possible it is recommended that records are kept of the length of time a cell line has been kept in culture. This is routinely achieved through the recording of passage numbers from the time of initial establishment. The use of continuous cell lines at low passage number (less than 50 passages from the time of immortalization) is recommended.

### *Genetic instability*

In general, the karyotype of cell lines is remarkably stable, provided that the culture conditions do not change. However, the chromosomal content of most continuous cell lines is both aneuploid (abnormal chromosome content) and heteroploid (variable chromosome content within the population). Many cancer cell lines have defects in p53 and other genes that monitor and repair DNA damage, resulting in an increased mutation frequency. Hence, the genotype of continuous cell lines can change with time and cell lines should not therefore be maintained for extended periods of time in continuous culture. Within a laboratory, provided culture conditions do not change, cell lines are remarkably stable. However, between laboratories, cell lines are subject to selective pressures, and genetic and phenotypic changes are often seen, for example in stocks of the human breast cancer cell line MCF-7.

© 2000 Cancer Research Campaign

In general, cell lines derived from normal tissue tend to have a normal chromosomal content, at least as far as can be judged from the karyotype. Most normal human cell lines will senesce and cease proliferation without major heritable changes in the genotype. In contrast, rodent cell lines, particularly mouse lines, become unstable and immortalize readily. Immortal cell lines, such as 3T3, can retain their normal (untransformed) growth characteristics provided that the recommended maintenance procedures are adhered to. In particular, they should not be allowed to become confluent, but should be subcultured from mid-log phase, and replaced regularly from frozen stock. They require constant monitoring to ensure that transformed variants, readily detected morphologically by their more retractile appearance and lack of contact inhibition, do not overgrow.

#### *Phenotypic instability*

Lack of expression of the differentiated properties of the cells of origin is a major recurrent problem. This can be due to selection of the wrong cell lineage in inappropriate culture conditions. For example, a disaggregated skin biopsy will ultimately give rise to a fibroblastic population which overgrows the epidermal keratinocytes, unless selective conditions are used. However, even under selective conditions, the need for propagation stimulates cell proliferation rather than differentiation. This process can either select undifferentiated cells or can lead to a loss of differentiated characteristics. In some cases, such as fibroblasts or endothelial cells, this is due to dedifferentiation, but in others, such as mammary epithelium, it is probably due to propagation and expansion of the progenitor cell compartment, which lacks the differentiated characteristics.

Examination of processes which depend on the expression of the in vivo phenotype, whether normal or neoplastic, may require modifications to culture conditions (e.g. high cell density, growth factors, low serum, position at the air–liquid interface, heterologous cell interaction, extracellular matrix) which usually are incompatible with cell proliferation. Hence different conditions need to be defined for culture of a cell line dependent on whether cell proliferation or cell differentiation is required.

To minimize genotypic and phenotypic variation of a cell line within and between laboratories, it should be expanded and frozen, and used to provide the seed stock for future work. Cells should be replaced from frozen stocks after a maximum of ten passages or 3 months continuous culture (whichever is the shorter). It is important and probably essential for comparative purposes that different laboratories using the same cell line should match their culture conditions as closely as possible.

### SECTION 5: CELL LINE DISTRIBUTION

### Introduction

Transferring a cell line between laboratories may involve transport within a city or between continents. Therefore, consideration will have to be given to the condition of the cells, the means of transport and the legal requirements. Cell lines may be transported either as growing cultures or as vials of frozen cells.

Within the UK and Western Europe, use of a courier service should ensure delivery within 48 h to most destinations. Delivery to most places outside of Europe should be possible within 96 h and this is compatible with sending growing cultures. However, it is impossible to guarantee that packages have remained under appropriate conditions (e.g. temperature, vibration-free) throughout the transport period. If frozen vials are sent, the fact that solid carbon dioxide remains within the package should be sufficient to ensure that transport conditions have been acceptable.

Some couriers will not accept boxes containing solid carbon dioxide for transportation and hence enquiries regarding this point should be made in advance.

### Transport containers

Cultures of adherent cells growing in flasks should not be sent with the usual volume of medium (e.g. 5 ml in a 25 $cm^2$ flask). Movement of the package during transport will result in excessive frothing and cell destruction. One method is to fill the flask completely with medium at the correct pH and hence totally exclude all gas. Disadvantages of this procedure are that the flask is heavy, there is a considerable volume of medium to leak if the flask is broken, and cultures may subsequently become infected because of medium around the neck and cap of the flask. An alternative method is to remove all except a few drops of medium from the flask, gas with the appropriate mixture, and seal the flask. The small volume of medium is sufficient to keep the cells moist but insufficient to allow frothing to occur, and cells remain viable for at least 72 h.

For suspension cultures or cells which grow as floating aggregates, 2 ml plastic freezing vials are suitable containers for transport. Cells in medium should be transferred to the vial in a volume of 1.0–1.5 ml and medium then added drop-by-drop to fill the vial before replacing the screw cap. Because of their size, such vials can be sent in small padded envelopes.

Insulated boxes suitable for transport of frozen vials of cells are used by various laboratory supply companies for distribution of frozen reagents. Those used by Amersham International for labile radioisotopes are of suitable thickness and dimensions. Such boxes typically have 5 cm thick walls with a central cavity of $15 \times 15 \times 15$ cm. This can be filled with solid carbon dioxide, which will maintain temperature for a maximum of 4 days.

### Practicalities

Experience dictates that adherence to the following points will increase the probability of successful transfer:

a. Communicate fully with the carrier and the recipient in advance. Be sure that they both know the collection time and the anticipated delivery time. Exchange telephone/fax numbers for use should problems arise
b. Make sure that the recipient knows what type of containers are being sent and the state of the cells. Make sure that they know what to do with the cells when they arrive, that they have the correct medium available, and that they are familiar with the growth characteristics of the cells
c. Ask the recipient to notify you when the cells arrive or when the cells have failed to arrive within a reasonable period
d. Send packages on a Monday to improve the chance of a weekday delivery
e. Ask the recipient to establish, as a high priority, their own frozen stock of the cells so that repeated transport is not needed.

*© 2000 Cancer Research Campaign*

### Regulations

Various regulations must be complied with when sending cells to other laboratories. These include legal requirements of various countries and regulations established by individual carriers. It is strongly recommended that full details of these are obtained before any transport is attempted. Regulations concerning the transport of potentially dangerous goods are published by the International Air Transport Association (IATA) and updated annually. Further information can be found on the IATA web site (http://www.iata.org/cargo/dg/dgr.htm).

It is beyond the scope of these Guidelines to spell out in detail the full regulations. However, the following points may be useful in providing general guidance:

*Within the UK*

The precise requirements for sending cells via the postal service in the UK are currently being revised. It is therefore recommended that the local Customer Service Centre of the Royal Mail must be contacted for advice prior to sending biological material by post.

*Import to the UK*

While there are few restrictions on the movement of cell cultures within the European Community, importation of certain animal cells from other countries into the UK requires a permit from the Ministry of Agriculture, Fisheries and Food (MAFF) at the Animal Health Disease Centre, Tolworth, Surbiton, Surrey KT6 7NF: This is particularly important for cells from agricultural species where there is a serious risk of importing non-endemic viruses (e.g. hog cholera virus in pig cell lines). It is strongly recommended that current regulations be checked, especially if there are doubts about the legality of the importation.

*Export from the UK*

Apart from the USA, few countries have specific regulations regarding the import of cell lines and hence sending cells abroad should not present major problems. However, if material is classified as ACDP category 2 or above (HMSO, 1995), special conditions apply and the sender must undergo formal training. It is recommended that the cell line(s) are sent by courier service and that the contents of the package must be clearly labelled on the ‘shipper’s declaration’ as ‘biological material for research purposes’.

*Sending cells to the USA*

Many people have experienced difficulties in sending cells to the USA. However, problems can be minimized by strict adherence to the following advice:

It is obligatory for the importation of cell lines or their products into the United States that an application is made for a Veterinary Permit from the US Department of Agriculture prior to shipment. To obtain a permit the forms VS 16-3 and VS 16–7 are requested by the recipient of the cells from the USDA (USDA, APHIS, Veterinary Services, National Center for Import and Export, 4700 River Road, Unit 40, Riverdale, MD 20737-1231, USA: Tel: +1-301-734-3277; Fax: +1-301-734-8226). The recipient must complete the forms and return them to the USDA address (faxed copies are accepted) with copies of other relevant information required. The main points of information required are:

a. The animal the product was derived from
b. The storage fluid (e.g. saline solution, bovine serum)
c. Full description of the culture including the origin of serum used in culture (other supporting documentation of the source of serum may be required and use of serum originating in the US can help)
d. The history of the product
e. The intended use
f. The country of origin
g. The recipient’s company name, contact name and telephone number
h. The correct temperature for storage.

Upon receipt of a satisfactorily completed form, the Department of Agriculture will issue a permit (VS form 16-16A). A copy of this permit should be taped to the outside of the package. This should expedite passage of the package through US customs.

### SECTION 6: TROUBLESHOOTING

Even with full attention to these Guidelines and/or other established rules of good practice, every laboratory will, from time to time, encounter problems ranging from widespread fungal contamination to quite subtle deviations from normal patterns of cell growth. When such problems occur, a logical and systematic approach should be taken to identifying and removing the causes.

Without good background information, assessment of a problem can be unnecessarily difficult or impossible. Careful logging of reagent batch numbers used to make each bottle of medium may often seem pedantic and time-consuming, but can prove invaluable when problems occur. Similarly, careful documentation of the normal behaviour of a cell line provides essential background information. This can include records of cell counts at subculture and occasional photographs of growing cultures.

The following general approach to troubleshooting may prove useful:

a. Once the existence of a problem is suspected, it is important to define its characteristics and inform all those who may be affected
b. If the nature of the problem is readily identified (e.g. a defective incubator), make sure by appropriate means that its existence is known (e.g. a large notice on the incubator) and that the person responsible is dealing with the problem
c. Less obvious problems will need a more comprehensive survey of the facts. This may be facilitated by a meeting of all those involved, as even apparently quite trivial observations may be relevant
d. Once the problem is identified, it should be possible to draw up a list of possible causes in order of probability
e. It is often useful to ask ‘what is new?’ in terms of reagents or procedures which may coincide with the problem. Be aware, however, of possible time displacements such as the effects of a minimally substandard medium batch only becoming manifest after several cell generations, with some cell lines being more sensitive than others
f. When switching to a new batch of any medium component (including serum), even though this has been batch-tested, retain a reasonable amount of the old batch for some period of time. This will allow head-to-head testing should problems arise when the new batch is introduced
g. With problems of deficient cell growth and/or unusual appearance, the problem may lie with the cells, the growth medium, the growth environment or some combination of these. Clues

© 2000 Cancer Research Campaign as to which of these to pursue first may come from which cell lines are affected (do they share an incubator?, do they have a common medium?)

h. If a particular cell line is affected, and tests for contamination (bacteria, mycoplasma) are negative, a vial of the cell line should be taken from frozen stock and the old and new cells tested head-to-head over several passages. If the old cells continue to do badly and the new cells grow normally, then the old cell stock should be discarded and the new stock used for future work. If both stocks do badly then the problem probably lies elsewhere. (Virtually all cell lines take a period of time to recover from being frozen, so this needs to be taken account of when comparing growth patterns.)
i. Where a problem with the medium appears to be present, a series of tests should be set up in which head-to-head growth comparisons are made in different media where only one medium component at a time is changed. Although serum and basic medium may be the most obvious sources of problems, other components including water, glutamine and antibiotics are also candidates
j. If a problem component is identified, the finding should be discussed with the supplier who may be able to state that the batch has been used by many other laboratories without problems or (occasionally and off-the-record) that there may be a problem with a batch
k. In the experience of many workers, problems sometimes are never satisfactorily solved, but the cells begin, after some time, to resume normal growth. Such problems may, however, recur and the combined experience of the first and second episodes may be helpful in further investigation.

### REFERENCES

Gartler SM (1967) Genetic markers as tracers in cell culture. Second Decennial Review Conference on Cell, Tissue and Organ Culture. *Natl Cancer Inst Monogr* **26**: 167–195

Gugel EA and Sanders ME (1986) Needle-stick transmission of human colonic adenocarcinoma. *New Engl J Med* **315**: 1487

Health and Safety Commission (1992) Guide to Genetically Modified Organisms (Contained Use) Regulations, 1992. HSE Guide, L29. HSE Books, Sudbury

Health and Safety Commission (1994) Control of Substances Hazardous to Health Regulations, 1994. Statutory Instrument No 3246, HSE Books, Sudbury

Health and Safety Executive (1990) Safety at Autoclaves, Guidance Note PM73, HSE Books, Sudbury

Health Services Advisory Committee (1992) Safe Disposal of Clinical Waste. HSE Books, Sudbury

HMSO (1992) British Standards Institute BS5726, Microbiological Safety Cabinets Parts 1–4. The Stationery Office Books, London

HMSO (1995) Advisory Committee on Dangerous Pathogens, 4th edition. Categorisation of biological agents according to hazard and categories of containment. The Stationery Office Books, London

HMSO (1996) Advisory Committee on Dangerous Pathogens. Protection against blood-borne infections: HIV and hepatitis. The Stationery Office Books, London

HMSO (1997) Rules and Guidance for Pharmaceutical Manufacturers and Distributors 1997, The Stationery Office Books, London

Hummeler K, Davidson WL, Henle W, LaBoccetta AC and Ruch HG (1989) Encephalomyelitis due to infection with Herpesvirus simiae (Herpes B virus). *New Engl J Med* **261**: 64–68

Lavappa KS (1978) Survey of ATCC stocks of human cell lines for HeLa contamination. *In Vitro* **14**: 469–475

Lloyd G and Jones N (1984) Infection of laboratory workers with hantavirus acquired from immunocytomas propagated in laboratory rats. *J Infect* **12**: 117–125.

McGarrity GJ, Vanaman V and Sarama J (1984) Cytogenetic effects of mycoplasmal infection of cell cultures: a review. *In Vitro* **20**: 1–18

Schaeffer WI (1990) Terminology associated with cell, tissue and organ culture, molecular biology and molecular genetics. *In Vitro Cell Dev Biol* **26**: 97–101

Schmid I, Nicholson JKA, Giorgi JV, et al (1997) Biosafety guidelines for sorting of unfixed cells. *Cytometry* **28**: 99–117

Southam CM (1958) Homotransplantation of human cell lines. *Bull NY Acad Med* **34**: 416–423.

Stacey GN and Doyle A (1997) Master Cell Banking. In: Procedures in Cell and Tissue Culture, 31A Standardisation, 31A1, Doyle A, Griffiths JB and Newell DG (eds). Wiley and Sons, Chichester

Tedder RS, Zuckerman MA, Goldstone AH, et al (1995) Hepatitis B transmission from a contaminated cryopreservation tank. *Lancet* **346**: 137–140

UKCCCR (1998) Guidelines for the Welfare of Animals in Experimental Neoplasia, 2nd edition. *Br J Cancer* **77**: 1–10.

### APPENDIX 1: DEFINITIONS OF TERMS FREQUENTLY USED IN TISSUE CULTURE

These guidelines use terms as defined by Schaeffer (1990).

A *primary culture* refers to a culture from the time of isolation until its first subculture.

A *cell line* is formed when a primary culture is subcultured. A cell line may be *finite* (survives for a fixed number of population doublings, usually around 40–60, before senescing and ceasing proliferation) or *continuous* (immortal, over 200 population doublings).

The use of the term ‘established’ for a *continuous* cell line is discouraged because it is ambiguous.

*Cell strains* are cell lines which have been purified by physical separation, selection or cloning, and which have specific defined characteristics, e.g. BHK-21-PyY, anchorage-independent cells cloned from the BHK-21 cell line following transformation with polyoma virus.

*Cloning* is the generation of a colony from a single cell, and subculture of such a colony would give rise to a cell strain. Because of potential confusion with molecular cloning, this term is probably better modified to *cell cloning*.

*Immortalization* is the indefinite extension of lifespan in culture, usually achieved by the introduction of a viral gene, but already acquired by some cancer cells.

*Transformation* is a heritable change involving an alteration in the genotype, usually subsequent to immortalization. It is best reserved to describe an alteration in growth characteristics associated with malignancy (anchorage independence, loss of contact inhibition and density limitation of cell proliferation, tumorigenesis in vivo).

### APPENDIX 2: PATIENT CONSENT FORM: POINTS TO CONSIDER

a. The donor is under stress and is being asked to help you. Your request is an additional factor which may add to that stress
b. The Patient Consent Form and associated Patient Information Sheet (necessary for most studies) should be written in concise and explicit language that anyone can easily understand, explaining clearly the need for the specimen, the overall objective of the research and why it is important (in lay terms)
c. The additional discomfort or inconvenience which will occur if the donor agrees to your request should be clearly explained
d. The donor should be told clearly that there is no obligation whatsoever to participate in your research

 © 2000 Cancer Research Campaign e. If the research may be exploited commercially, the donor should be told clearly what financial benefit might be gained from the research and a waiver to commercial rights should be requested
f. The donor should be told that the research has been approved by the local Ethics Committee (give date and reference)
g. All forms should be marked Confidential
h. It should be made clear that confidentiality will be assured, but if not (e.g. familial studies) indicate who will have access to the clinical data and how access will be controlled
i. Fully informed consent means that the person should have access to all information relating to the use of the specimen provided. The details may be covered in a Patient Information Sheet, but the name(s) and phone number(s) of investigators should be included on the form and sheet, so that the donor can obtain further information if needed
j. The information sheet and consent form must be printed on official headed notepaper.
k. Consent forms should address the following questions:

Have you read the information sheet about this study?
Have you had an opportunity to ask questions and discuss the study?
Have you received satisfactory answers to all your questions?
Have you received enough information about this study?
Which doctor have you spoken to about this study?
Do you understand that you are free to withdraw from this study (i) at any time, (ii) without giving a reason and (iii) without affecting your future medical care?

### APPENDIX 3: CELL CULTURE BANKS

| Collection | Address | Web site |
|---|---|---|
| American Type Culture Collection (ATCC) | 10801 University Blvd, Manassas, VA 20108-1549, USA | http://www.atcc.org |
| Coriell Cell Repository | 401 Haddon Avenue, Camden, NJ 08103, USA | http://locus.umdnj.edu/ccr |
| Deutsche Sammlung von Mikroorganismen und Zellkulturen (DSMZ) | Mascheroder Weg 1b, D-38124 Braunschweig, Germany | http://www.dsmz.de |
| European Collection of Animal Cell Cultures (ECACC) | CAMR, Salisbury, Wilts SP4 0JG, UK | http://www.camr.org.uk |
| Japanese Collection of Research Bioresources (JCRB) | 1-1-43 Hoen-Zaka, Chuo-Ku, Osaka 540, Japan | http://cellbank.nihs.go.jp |
| RIKEN Gene Bank | 3-1-1 Koyadai, Tsukuba Science City, Ibaraki 305, Japan | http://www.rtc.riken.go.jp |

### APPENDIX 4: METHODS FOR CELL LINE IDENTIFICATION

| Technique | Advantages | Disadvantages |
|---|---|---|
| DNA fingerprinting Multilocus repeat sequences | Specific identification of origin. Can be used to measure genetic relatedness | Requires high degree of technical reproducibility. No gene identification. |
| DNA fingerprinting Random primers | Rapid with high degree of specificity | Requires careful validation for reproducibility. No gene identification. |
| DNA profiling by PCR of single loci | Rapid, reproducible and easy to read. Allele frequencies known | Several loci must be analysed for unequivocal identification within species. Primers specific for each species need to be included. |
| Isozyme typing | Rapid identification of species. Standardized kits available | Low sensitivity. Several isozymes must be analysed for unequivocal identification within species |
| Karyotyping | Detection of marker chromosomes may allow unique identification of individual cell lines | Requires relatively high degree of skill in technique and interpretation |

© 2000 Cancer Research Campaign

### APPENDIX 5: MYCOPLASMA DETECTION METHODS

| Method | Sensitivity | Advantages | Disadvantages |
|---|---|---|---|
| Direct DNA stain (e.g. Hoechst 33258) | Low | Rapid, cheap | Can be difficult to interpret |
| Indirect DNA stain on indicator cells (e.g. 3T3) | High | Amplifies contamination, so easy to interpret | Indirect and thus more time-consuming |
| Broth and agar culture | High | Sensitive | Relatively slow and may require expert interpretation |
| ELISA | Moderate | Rapid | Limited range of species detected |
| PCR | High | Rapid | Restriction digest analysis of PCR product or multiplex PCR may be needed to distinguish species |

Positive and negative controls should be included

### SUMMARY

### Section 1: Derivation of a new cell line

1A Obtain permission from the Local Research Ethics Committee (human material) or Animal Welfare Committee (animal material).
1B Clarify ownership and ethical issues, bearing in mind possible future commercial exploitation.
1C Only transfer the cell line to other laboratories after completion of a ‘Materials Transfer Agreement’.
1D Document the clinical details of the donor.
1E Store tumour and normal tissue and use to confirm cell line origin by DNA fingerprinting or profiling.
1F Document all reagents, culture conditions and passage dates during derivation of the cell line.
1G Ensure that the cell line has a unique, unambiguous designation.
1H Freeze down an initial bank of cells as soon as possible.

### Section 2: Cell line acquisition

2A Cell lines being brought into the laboratory should go through a quarantine procedure.
2B Characterize the new line to confirm its identity and freedom from infection.
2C Cell lines acquired from established culture collections are unlikely to carry bacterial, fungal or mycoplasma contamination, but in most cases there is no guarantee that the cell line is not cross-contaminated.
2D Freeze down a bank of cells as soon as possible.

### Section 3: Cell line practice

3A Ensure that you are familiar with local Safety Rules as established by the institution’s Biological Safety Officer.
3B Handle clinical specimens as potentially infected with HIV, hepatitis or other hazardous agents.
3C Establish correct disposal routes for all types of laboratory waste before starting a procedure.
3D Ensure that staff have received adequate training before their work commences.
3E Establish a Master Cell Bank and a Working Cell Bank for each cell line in use. Determine rules of access to these cell stocks.
3F Replace working cell stocks from the bank at regular intervals (ten passages or at 3 months, whichever is shorter).
3G Purchase media and reagents (especially serum) from established sources.
3H Keep medium preparation entirely separate from procedures involving living cells.
3I Keep full and careful records of all batch numbers of reagents and media.
3J Establish ‘Standard Operating Procedures’ for all routine laboratory procedures.
3K Ensure that all items of laboratory equipment (cabinets, incubators, autoclaves, water filtration units) are properly serviced and are working within prescribed limits.
3L Inspect the cells under an inverted phase microscope before use. For routine culture, inspect cells regularly (at 2- to 3-day intervals as a minimum).

### Section 4: Cell line problems

4A Handle cell lines entirely separately so as to minimize the risk of cross-contamination.
4B Do not add antibiotics to the culture medium.
4C Carry out regular tests for mycoplasma contamination, especially immediately before establishing a frozen stock. Subsequently check for contamination on a thawed sample from the frozen stock.

*© 2000 Cancer Research Campaign*

4D Cells carrying infection with bacteria, fungi or mycoplasma should be discarded unless some unique property requires that ‘clean-up’ be attempted. Such procedures are sometimes effective, but a long time will be needed to ensure that contamination has successfully been eliminated.

4E Be aware of the possibility of ‘phenotypic drift’ during repeat passaging of cell lines. Establish a pattern of routine reversion to frozen stock so as to minimize the effect (see 3F).

### Section 5: Cell line distribution

5A Only transfer cells after full discussion of all details with the recipient.

5B Ensure that the recipient has all the necessary information to handle the cells correctly following receipt.

5C Determine the regulations of individual courier services before attempting to send cells outside the UK.

5D Only send cells to the USA after obtaining clearance from the US Department of Agriculture.

© 2000 Cancer Research Campaign

## References

[PDF_01288] Gartler S. M. (1967). Genetic markers as tracers in cell culture.. Natl Cancer Inst Monogr.

[PDF_01291] Gugel E. A., Sanders M. E. (1986). Needle-stick transmission of human colonic adenocarcinoma.. N Engl J Med.

[PDF_01312] HUMMELER K., DAVIDSON W. L., HENLE W., LABOCCETTA A. C., RUCH H. G. (1959). Encephalomyelitis due to infection with Herpesvirus simiae (herpes B virus); a report of two fatal, laboratory-acquired cases.. N Engl J Med.

[PDF_01315] Lavappa K. S. (1978). Survey of ATCC stocks of human cell lines for HeLa contamination.. In Vitro.

[PDF_01317] Lloyd G., Jones N. (1986). Infection of laboratory workers with hantavirus acquired from immunocytomas propagated in laboratory rats.. J Infect.

[PDF_01320] McGarrity G. J., Vanaman V., Sarama J. (1984). Cytogenetic effects of mycoplasmal infection of cell cultures: a review.. In Vitro.

[PDF_01327] SOUTHAM C. M. (1958). Homotransplantation of human cell lines.. Bull N Y Acad Med.

[PDF_01322] Schaeffer W. I. (1990). Terminology associated with cell, tissue, and organ culture, molecular biology, and molecular genetics. Tissue Culture Association Terminology Committee.. In Vitro Cell Dev Biol.

[PDF_01325] Schmid I., Nicholson J. K., Giorgi J. V., Janossy G., Kunkl A., Lopez P. A., Perfetto S., Seamer L. C., Dean P. N. (1997). Biosafety guidelines for sorting of unfixed cells.. Cytometry.

[PDF_01332] Tedder R. S., Zuckerman M. A., Goldstone A. H., Hawkins A. E., Fielding A., Briggs E. M., Irwin D., Blair S., Gorman A. M., Patterson K. G. (1995). Hepatitis B transmission from contaminated cryopreservation tank.. Lancet.

